# Amides Derived from Vanillic Acid: Coupling Reactions, Antimicrobial Evaluation, and Molecular Docking

**DOI:** 10.1155/2019/9209676

**Published:** 2019-04-15

**Authors:** Ana Júlia de Morais Santos Oliveira, Ricardo Dias de Castro, Hilzeth de Luna Freire Pessôa, Abdul Wadood, Damião Pergentino de Sousa

**Affiliations:** ^1^Laboratory of Pharmaceutical Chemistry, Department of Pharmaceutical Sciences, Federal University of Paraíba, 58051-900 João Pessoa, PB, Brazil; ^2^Laboratory of Experimental Pharmacology and Cell Culture of the Health Sciences Center, Federal University of Paraíba, 58051-900 João Pessoa, PB, Brazil; ^3^Laboratory of Toxicological Tests-Labetox, Federal University of Paraíba, 58051-900 João Pessoa, PB, Brazil; ^4^Abdul Wali Khan University Mardan, 23200 Mardan, Pakistan

## Abstract

A series of amides derived from vanillic acid were obtained by coupling reactions using PyBOP ((Benzotriazol-1-yloxy)tripyrrolidinophosphonium hexafluorophosphate) and DCC (Dicyclohexylcarbodiimide) coupling reagents. These were submitted to biological evaluation for species of* Candida*,* Staphylococcus*, and* Pseudomonas*. The microdilution method in broth was used for the antimicrobial testing to determine the Minimum Inhibitory Concentration (MIC) and to verify the likely mechanism of action for antifungal activity. The ten amides were obtained with yields ranging from 28.81 to 86.44%, and three compounds were novel. In the antibacterial evaluation, the amides (in their greatest concentrations) were bioactive against* Staphylococcus aureus *strain ATCC 25925. Meanwhile, all of the tested amides presented antifungal activity against at least one strain. The amide with best antifungal profile was compound** 7**, which featured an MIC of 0.46 *μ*mol/mL, and a mechanism of action involving the plasma membrane and fungal cell wall. The presence of a methyl group in the* para* position of the aromatic ring is suggested which enhances the activity of the compound against fungi. Docking studies of the ten compounds using the protein 14*α*-demethylase as a biological target were also performed. The biological results presented good correlation with molecular docking studies demonstrating that a possible site of antifungal action for bioactive amides is the enzyme 14*α*-demethylase.

## 1. Introduction

Microorganisms are routinely associated with diverse serious diseases in a wide range of infections. However, some microorganisms contribute to the organism in terms of maintenance and balance, living in harmony with the host [1, 2]. Within the context of infections caused by microorganisms, we highlight the presence of the Gram positive bacterium* Staphylococcus aureus* in the normal body microbiota, which can act pathogenically in wide ranges, from the most superficial to the most disseminated and severe infections [3, 4].

In addition to bacteria, the eukaryotes such as fungi are generally considered to be harmless. However, principally affecting immunocompromised individuals, fungal infections contribute to the increased prevalence of hospital-acquired infections, having high impacts on mortality rates [5]. Treatments of fungal and bacterial infections often use antimicrobial agents from natural or synthetic sources which act on microorganisms by inhibiting growth or causing extermination [6]. In the last decade a considerable increase in the prevalence of antimicrobial resistance associated with prolonged treatment times, greater toxicity, and higher costs has been observed [7]. Antimicrobial resistance itself emphasizes the need for further study to obtain new pharmacologically effective and less toxic substances.

**Figure 1:**
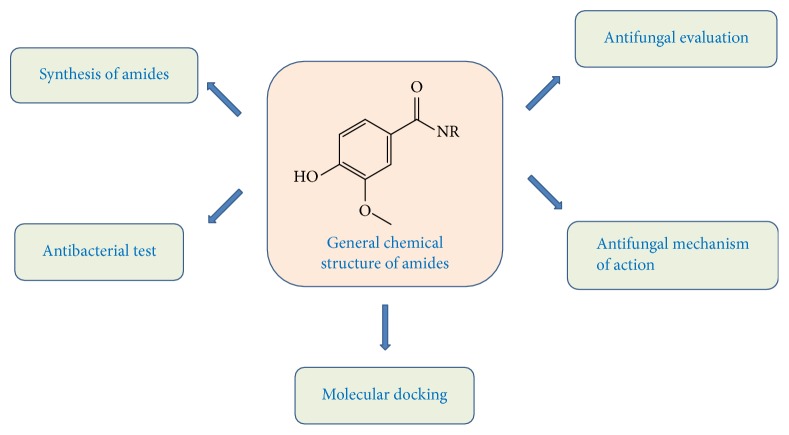
Schematic representation of the study using amides derived from vanillic acid.

Phenolic compounds are widely distributed in nature, participating in the compositions of vegetables and fruits, and recognized as having antitumor, antimicrobial, and cardiovascular preventive and antidegenerative activities among others [8–10]. From this group, there are the phenolic acids (cinnamic and benzoic acid derivatives) and coumarins [11]. The benzoic acid derivatives such as gentisic, vanillic, and* p*-hydroxybenzoic acid present antimicrobial properties against many bacterial and fungal strains [12, 13]. Thus, the objective of this study was to prepare derivative vanillic acid amides and assess their antibacterial and antifungal activities to obtain a derivative with a better antimicrobial profile and as well perform molecular docking of the ten compounds obtained (Figure 1), evidencing potential biological activity when targeting the protein 14*α*-demethylase.

## 2. Experimental Section

### 2.1. Chemistry

#### 2.1.1. Materials

Compounds purification was performed using column chromatography, silica gel 60, ART 7734 MERCK with a Hex:EtOAc solvent gradient, accompanied by thin-layer analytic chromatography on silica gel 60 F254, MERCK, exposed to ultraviolet irradiation at two wavelengths (254 and 366 nm) with a MINERALIGHT device, and also using plates revealed by 5% H_2_SO_4_ solution in ethanol, plotted on heating plates. The infrared spectroscopy was performed by FTIR spectrophotometer, model* Irprestige-21*, Shimadzu, potassium bromide (KBr) pills, and frequency measurement in cm^−1^. The ^13^C and ^1^H NMR spectra were obtained on Varian MERCURY machines operating at 400 and 100 MHz and at 500 and 125 MHz for ^1^H and ^13^C, respectively. CDCl_3_ or DMSO-d_6_ solvent was used. Tetramethylsilane (TMS) was used as the internal standard. Chemical deviations (d) were measured in parts per million (ppm) and the coupling constants (J) in Hz. The high resolution mass spectrometry was performed for the original compounds, using a TOF/TOF Ultraflex II mass spectrometer equipped with a high performance solid state laser (*λ* = 355 nm) and a reflector. The system was operated by the FlexControl 2.4 software package (Bruker Daltonics GmbsH, Bremen-Germany).

#### 2.1.2. Vanillic Acid Derived Amides (**1-6**)

Vanillic acid (0.59 mmol), triethylamine (0.59 mmol), dimethylformamide (0.59 mmol), and amine (0.59 mmol) were mixed and a solution of PyBOP (0.59 mmol) dissolved in 1.2 mL of dichloromethane (CH_2_Cl_2_) was added. The reaction was kept in an ice bath for 30 minutes under constant magnetic stirring and then was brought to room temperature for 3 to 7 hours. The solvent was evaporated under reduced pressure and the residue was treated with distilled water (10 mL) and then extracted with ethyl acetate (3 x 10 mL). The organic phase was collected and treated with 1N (10 mL) hydrochloric acid solution, sodium bicarbonate solution 5% (10 mL), and distilled water (10 mL). The organic phase was dried with anhydrous Na_2_SO_4_ and the solvent was evaporated under reduced pressure. The residue was purified by silica gel column chromatography (Hexane-EtOAc, 6:4 to 2:8) to obtain the described compounds [14].

#### 2.1.3. Vanillic Acid Derived Amides (**7-10**)

Vanillic acid (0.59 mmol), along with 4-dimethylaminopyridine (0.059 mmol), followed by amine (0.59 mmol) were mixed and added to a solution of dicyclohexylcarbodiimide (0.59 mmol) dissolved in 3 mL of dichloromethane (CH_2_Cl_2_). The solution was stirred for 24 to 48 hours at room temperature. The solvent was evaporated under reduced pressure and the residue was treated with distilled water (10 mL) and extracted with ethyl acetate (3 x 10 mL). The organic phase was collected and treated with 1N hydrochloric acid solution (10 mL), then the sodium bicarbonate solution 5% (10 mL), and distilled water (10 mL). The organic phase was dried with anhydrous Na_2_SO_4_ and the solvent was evaporated under reduced pressure. The residue was purified by silica gel column chromatography (Hexane-EtOAc, 6:4 to 2:8) to obtain the described compounds [15].

*4-Hydroxy-N-isobutyl-3-methoxybenzamide (* ***1***). Dark brown oil, 28.81% yield; IR *ʋ*max (KBr, cm^−1^): 3342, 2958, 2927, 1631, 1589, 1552 and 1512; ^1^H NMR (500 MHz, CDCl_3_) *δ*_H_ 7.46 (*d, J =* 1.9 Hz, 1H, H-2), 7.18 (*dd, J =* 8.2, 2.0 Hz, 1H, H-6), 6.91 (*d, J =* 8.2 Hz, 1H, H-5), 6.02 (*s*, 1H, OH), 3.93 (*s*, 3H, OCH_**3**_), 3.27 (*t, J =* 6.3 Hz, 2H, H-1′), 1.88 (*m, *1H, H-2′), 0.97 (*d, J =* 6.7 Hz, 6H, H-3′, H-4′); ^13^C NMR (125 MHz, CDCl_3_): *δ*_C_ 167.50 (C=O), 148.81 (C-4), 146.71 (C-3), 126.90 (C-1), 119.51 (C-6), 114.07 (C-5), 111.02 (C-2), 56.01 (O**C**H_3_), 47.32 (C-1′), 28.44 (C-2′), 20.34 (C-3′) [13].

*(4-Hydroxy-3-methoxyphenyl) (Pyrrolidine-1-yl) Methanone (* ***2***). Dark yellow oil, 86.44 % yield; IR *δ*_C_*ʋ*max (KBr, cm^−1^): 3184, 2970, 2877, 1662, 1585, 1570 and 1517; ^1^H NMR (500 MHz, CDCl_3_): *δ*_H_ 7.11 (*d, J =* 1.8 Hz, 1H, H-2), 7.02 (*dd, J =* 8.1, 1.9 Hz, 1H, H-6), 6.87 (*d, J =* 8.2 Hz, 1H, H-5), 3.86 (*s*, 3H, OCH_**3**_), 3.61 (*t*,* J =* 5.9 Hz, 2H, H-1′), 3.48 (*t, J =* 5.4 Hz, 2H, H-2′), 1.96 – 1.80 (*m*, 4H, H-3′, H-4′); ^13^C NMR (125 MHz, CDCl_3_): *δ*_C_ 169.39 (C=O), 147.43 (C-4), 146.36 (C-3), 128.60 (C-1), 120.72 (C-6), 113.73 (C-5), 110.67 (C-2), 55.85 (OCH_3_), 49.89 (C-1′), 46.30 (C-2′), 26.32 (C-3′), 24.26 (C-4′) [13].

*N-Cyclohexyl 4-Hydroxy-3-methoxybenzamide (* ***3***). White crystalline solid, 51.67 % yield, m.p 210-211°C; IR *ʋ*max (KBr, cm^−1^): 3313, 2924, 2852, 1633, 1583, 1546 and 1510; ^1^H NMR (400 MHz, DMSO-d_6_): *δ*_H_ 7.29 (*q, J =* 2.0 Hz, 1H, H-2), 7.23 (*dd, J =* 8.2, 2.0 Hz, 1H, H-6), 6.68 (*d, J =* 8.2 Hz, 1H, H-5), 3.69 (*s*, 3H, OCH_**3**_), 2.39 (*q, J = *1.8 Hz, 2H, H-1′), 1.73 – 1.41 (*m*, 6H, H-2′, H-4′, H-6′), 1.24 – 1.11 (*m*, 4H, H3′, H-5′); ^13^C NMR (100 MHz, DMSO-d_6_): *δ*_C_ 164.72 (C=O), 149.11 (C-4), 147.02 (C-3), 125.65 (C-1), 120.50 (C-6), 114.53 (C-5), 111.42 (C-2), 55.50 (OCH_3_), 48.10 (C-1′), 32.40 (C-2′, C-6′), 25.13 (C-4′), 24.86 (C-3′, C-5′) [13].

*4-Hydroxy-3-methoxy-N-phenylbenzamide (* ***4***). Brown oil, 68.75% yield; IR *ʋ*max KBr, cm^−1^): 3300, 2966, 2935, 1645, 1597, 1533 and 1514; ^1^H NMR (400 MHz, CDCl_3_): *δ*_H_ 8.00 (*s*, 1H, NH), 7.63 (*dd, J =* 8.6, 1.1 Hz, 2H, H-2′, H-6′), 7.50 (*d, J =* 2.0 Hz, 1H, H-2), 7.36 (*dt, J =* 3.0, 1.9 Hz, 2H, H-3′, H-5′), 7.33 (*t, J =* 2.3 Hz, 1H, H-6), 7.16 – 7.10 (*m*, 1H, H-4′), 6.93 (*d, J = *8.2 Hz, 1H, H-5), 6.16 (*s*, 1H, OH), 3.90 (*s*, 3H, OCH_**3**_); ^13^C NMR (100 MHz, CDCl_3_): *δ*_C_ 165.60 (C=O), 149.23 (C-4), 146.95 (C-3), 138.20 (C-1′), 129.88 (C-6′), 129.17 (C-3′, C-5′), 127.10 (C-1), 124.53 (C-2′, C-4′), 120.43 (C-6), 114.14 (C-5), 110.72 (C-2), 56.27 (O**C**H_3_) [13].

*N-Benzyl-4-hydroxy-3-methoxybenzamide (* ***5***). Amorphous white solid, 66.54% yield, m.p 154-155°C; IR *ʋ*max (KBr, cm^−1^): 3286, 2960, 2852, 1633, 1583, 1548 and 1510; ^1^H NMR (500 MHz, CDCl_3_): *δ*_H_ 7.48 (*d, J =* 1.9 Hz, 1H, H-2), 7.34* (d, J =* 4.4 Hz, 4H, H-2′, H-3′, H-5′, H-6′), 7.30 – 7.26 (*m*, 1H, H-4′), 7.21 (*dd, J =* 8.2, 1.9 Hz, 1H, H-6), 6.89 (*d, J =* 8.2 Hz, 1H, H-5), 4.61 (*d, J =* 5.7 Hz, 2H, H-7′), 3.90 (*s*, 3H, OCH_**3**_); ^13^C NMR (125 MHz, CDCl_3_): *δ*_C_ 167.13 (C=O), 148.98 (C-4), 146.83 (C-3), 138.48 (C-1′), 128.87 (C-3′), 127.99 (C-5′), 127.90 (C-2′), 127.80 (C-6′), 127.67 (C-4′), 126.60 (C-1), 119.88 (C-6), 114.09 (C-5), 110.70 (C-2), 56.21 (OCH_3_), 44.27 (C-7′) [13].

*N-(4-Fluorobenzyl)-4-hydroxy-3-methoxybenzamide (* ***6***). Amorphous white solid, 30.41% yield, m.p 161-165°C; IR *ʋ*max (KBr, cm^−1^): 3115, 2939, 2829, 1641, 1591, 1560 and 1512; ^1^H NMR (500 MHz, DMSO-d_6_): *δ*_H_ 8.77 (*s*, 1H, NH), 7.44 (*d, J =* 2,0 Hz, 1H, H-2), 7.38 (*dd*,* J* = 8.3, 2.1 Hz, 1H, H-6), 7.32 – 7.29 (*m*, 2H, H-2′, H-6′), 7.11 (*t*,* J* = 9.0 Hz, 2H, H-3′, H-5′), 6.79 (*d*,* J* = 8.2 Hz, 1H, H-5), 4.41 (*d, J* = 6.0 Hz, 2H, H-7′), 3.78 (*s*, 3H, OCH_**3**_); ^13^C NMR (125 MHz, DMSO-d_6_): *δ*_C_ 166.05 (C=O), 161.25 (*d*, ^1^*J*_*C-F*_ = 237.5 Hz, C-4′-F), 149.53 (C-4), 147.25 (C-3), 136.22 (C-1′), 129.20 (C-2′), 129.14 (C-6′), 125.23 (C-1), 120.81 (C-6), 115.01 (C-5), 114.84 (C-3′, C-5′), 111.33 (C-2), 55.93 (OCH_3_), 41.98 (C-7′) [13].

*4-Hydroxy-3-methoxy-N-(4-methylbenzyl)benzamide (* ***7***). Yellow amorphous solid, 30.50 % yield, m.p 162-164°C; IR *ʋ*max (KBr, cm^−1^): 3273, 3016, 2918, 1631, 1583, 1546 and 1508; ^1^H NMR (400 MHz, CDCl_3_): *δ*_H_ 7.45 (*d, J =* 1.9 Hz, 1H, H-2), 7.24 – 7.19 (*m*, 3H, H-5, H-2′, H-6′), 7.13 (*d, J =* 7.6 Hz, 2H, H-3′, H-5′), 6.87 (*dd, J =* 8.2, 2.6 Hz, 1H, H-6), 4.55 (*d, J =* 5.2 Hz, 2H, H-7′), 3.86 (*s, J =* 10.3 Hz, 3H, OCH_**3**_), 2.32 (*s*, 3H, CH_**3**_); ^13^C NMR (100 MHz, CDCl_3_): *δ*_C_ 167.21 (C=O), 148.98 (C-4), 146.83 (C-3), 137.29 (C-4′), 135.37 (C-1′), 129.46 (C-3′, C-5′), 127.96 (C-4′, C-6′), 126.46 (C-1), 119.92 (C-6), 114.13 (C-5), 110.66 (C-2), 56.09 (OCH_3_), 43.98 (C-7′), 21.17 (CH_3_) [13].

*4-Hydroxy-3-methoxy-N-(4-methoxybenzyl)benzamide (* ***8***). Brown oil, 49.90% yield; IR *ʋ*max (KBr, cm^−1^): 3321, 2935, 2837, 1629, 1585, 1550 and 1514; ^1^H NMR (400 MHz, CDCl_3_): *δ*_H_ 7.49 (*d, J =* 2.0 Hz, 1H, H-2), 7.30 – 7.27 (*m*, 2H, H-3′, H-5′), 7.22 (*dd, J =* 8.3, 2.0 Hz, 1H, H-6), 6.92 – 6.87 (*m*, 3H, H-5, H-2′,H-6′), 4.57 (*d, J =* 5.6 Hz, 2H, H-7′), 3.92 (s, 3H,* m*-OCH_**3**_), 3.81 (s, 3H,* p*- OCH_**3**_); ^13^C NMR (100 MHz, CDCl_3_): *δ*_C_ 167.16 (C=O), 159.47 (C-4′), 149.04 (C-4), 147.25 (C-3), 130.55 (C-1′), 129.21 (C-2′, C-6′), 126.78 (C-1), 122.94 (C-6), 120.12 (C-5), 114.22 (C-3′, C-5′), 110.69 (C-2), 56.36 (C-3-O**C**H_3_), 55.48 (C-4′- O**C**H_3_), 43.93 (C-7′) [13]; EMAR (MALDI) calculated for C_16_H_17_NO_4_ [M]^+^: 287.3082; found 287.3089.

*N-(3,4-dimetoxibenzil)-4-hydroxy-3-methoxybenzamide (* ***9***). Yellow oil, 30.44% yield; IR *ʋ*max (KBr, cm^−1^): 3379, 2983, 2837, 1629, 1589, 1550 and 1514; ^1^H NMR (500 MHz, CDCl_3_): *δ*_H_ 7.47 (*d, J =* 1.8 Hz, 1H, H-2), 7.20 (*dd, J =* 6.3, 1.9 Hz, 1H, C-6), 6.88 (*q, J =* 6.2, 2.1 Hz, 3H, H-2′, H-3′, H-6′), 6.81 (*d, J =* 7,8 Hz, 1H, H-5), 5.29 (*s*, 1H, OH), 4.54 (*d, J =* 5.6 Hz, 2H, H-7′), *∗*3.91 (*s*, 3H,* p*-OCH_3_), *∗*3.85 (*d*,* J* = 3.1 Hz, 6H,* m*-OCH_3_); ^13^C NMR (101 MHz, CDCl_3_): *δ*_C_ 167.03 (C=O), 149.33 (C-4), 148.96 (C-5′), 148.68 (C-3), 146.83 (C-4′), 131.06 (C-1′), 126.64 (C-1), 120.35 (C-2′), 119.84 (C-6), 114.07 (C-5), 111.44 (C-3′), 111.53 (C-2), 110.67 (C-6′), *∗*56.21 (C-3-O**C**H_3_), *∗*55.08 (C-3′-O**C**H_3_), *∗*55,80 (C-4′-O**C**H_3_), 44.15 (C-7′) [13]; *∗*Interchangeable signals. EMAR (MALDI) calculated for C_17_H_19_NO_5_ [M]^+^: 317.3337; found 317.3380.

*N-(Benzo[d][1,3]dioxol-5-yl-methyl)-4-hydroxy-3-methoxybenzamide (* ***10***). Yellow amorphous solid, 29.25% yield, m.p 130-13°C; IR *ʋ*max (KBr, cm^−1^): 3111, 2935, 1635, 1583, 1554 and 1506; ^1^H NMR (400 MHz, CDCl_3_): *δ*_H_ 7.44 (*d, J =* 2.0 Hz, 1H, H-2), 7.21 (*dd, J =* 6.2, 2.0 Hz, 1H, H-6), 6.85 (*d, J =* 8.2 Hz, 1H, H-5), 6.80 (*d, J =* 1.4 Hz, 1H, H-6′), 6.76 (*dd, J =* 6.3, 1.5 Hz, 1H, H-2′), 6.71 (*d, J =* 7.9 Hz, 1H, H-3′), 5.90 (*s*, 2H, H-8′), 5.28 (*s*, 1H, OH), 4.47 (*d, J =* 5.7 Hz, 2H, H-7′); 3.84 (*s*, 3H, OCH_3_); ^13^C NMR (100 MHz, CDCl_3_): *δ*_C_ 167.09 (C=O), 149.01 (C-4), 148.05 (C-3), 147.12 (C-4′), 146.84 (C-5′), 132.35 (C-1′), 126.52 (C-1), 121.27 (C-2′), 119.88 (C-6), 114.10 (C-5), 110.68 (C-2), 108.60 (C-6′), 108.44 (C-3′), 101.18 (C-8′), 56.19 (O**C**H_3_), 44.08 (C-7′) [13]; EMAR (MALDI) calculated for C_16_H_15_NO_5_ [M]^+^: 301.2914; found 301.2901.

### 2.2. Antifungal Activity

Antifungal activity evaluations were performed using the following strains:* Candida albicans* ATCC 90028;* Candida glabrata* ATCC 90030;* Candida krusei *ATCC 34125, and* Candida guilliermondii* 207. The culture medium used for maintenance of the microorganisms was Agar Sabouraud Dextrose (ASD). For preparation of the inoculants used in the experiments, the yeasts were cultivated in Sabouraud Dextrose Broth (SDB) and were incubated for 24 hours at 35 ± 2°C. Inoculants were adjusted to a final concentration of 2.5 x 10^3^ CFU/mL [16].

#### 2.2.1. Determination of Minimum Inhibitory Concentration (MIC) of the Tested Compounds

The MIC was determined using the microdilution technique, as previously described by the CLSI adapted [16]. The compounds were subjected to microdilution technique in 96-well plates. The samples were dissolved with DMSO and sterile distilled water (to 1.0 mL) obtaining an initial solution of 4000 *μ*g/mL. Sabouraud Dextrose Broth (SDB) 100 *μ*L was distributed to the 96 wells of the microdilution plates, with U background. Then, 100 *μ*L of compound solutions was distributed to the first well line of the plate. In a serial dilution with a ratio of two, it provides final concentrations that ranged from 1000 *μ*g/mL to 7.8 *μ*g/mL. We then added 100 *μ*L of fungal strain inoculum to each well of the plates. The culture sterility medium, the evaluated substances, and microbial growth were executed in parallel. The plates were closed and subjected to a temperature of 35 ± 2°C for 24 hours. The TTC at 1% (2,3,5-triphenyl tetrazolium chloride, Sigma-Aldrich®) dye was added to each well in order to confirm the presence of viable microorganisms [17]. The MIC was defined as the lowest concentration of the test substance inhibiting visible microbial growth.

#### 2.2.2. Determination of Minimum Fungicide Concentration (MFC) of the Tested Compounds

The Minimum Fungicide Concentration (MFC) of the compounds was obtained after MIC interpretation. Three (3) 30 *μ*L aliquots of supernatant were removed from the wells (where complete fungal growth inhibition was analyzed) and allocated to Petri dishes containing 15 mL of Sabouraud Dextrose. The plates were incubated at 35 ± 2°C for 24 hours to visual count colony forming units [18, 19].

#### 2.2.3. Antifungal Mechanism of Action for the Amides

*(1) Sorbitol Assay*. Microdilution technique was performed in the presence of sorbitol (D-sorbitol, anhydrous) (INLAB laboratory), to determine the mode of action of amide** 7** on the cell wall of* C. guilliermondii* 207. For this test, inoculum was prepared with sorbitol to a final concentration of 0.8 M. The plates were incubated at 35 ± 2°C, and readings were taken at 24 hours and 48 hours after incubation. Caspofungin was used as a positive control at an initial concentration of 5 mg/mL [20–22].

*(2) Ergosterol Assay*. The testing was performed using the microdilution technique, as described earlier, in the presence of exogenous ergosterol (Sigma-Aldrich) in increasing concentrations (100, 200, and 400) *μ*g/mL, using Nystatin as a positive control. The plates were incubated at 35 ± 2°C and the readings were performed at 24 and 48 hours. Tween 80 and 96% ethanol were used for preparation of the ergosterol [20–24].

### 2.3. Antibacterial Activity

Testing to evaluate the antibacterial activity of the amides was executed using Gram-positive and Gram-negative bacteria coming from the American Type Culture Collection (ATCC) and from clinical origin:* Pseudomonas aeruginosa *ATCC 8027;* Pseudomonas aeruginosa* 102;* Staphylococcus aureus* ATCC 25925, and* Staphylococcus aureus* 47. These were grown in a medium consisting of yeast extract (Himedia) 5 g, peptone (Merck) 10 g, NaCl (Merck) 5 g, KH_2_PO_4_ (Merck) 1.5 g, and NaHPO_4_.12.H_2_O (Merck) 9 g solubilized in 1 L of distilled water and sterilized in an autoclave at 121°C, at 1 atm, for 20 minutes. The inoculum was adjusted according to the standard 0.5 McFarland range, containing 1.5 x 10^8^ CFU/mL [25].

#### 2.3.1. Determination of Minimum Inhibitory Concentration (MIC) for Bacterial Samples

Determination of the MIC was executed by means of microdilution technique using one 96-well plate for each of the tested strains. A 10 mg/mL solution of the compounds was prepared using 25% DMSO in water. For this, 100 *μ*L of culture medium was sewn into all wells, except for the 1st and 2nd columns which received 150 *μ*L. Then 50 *μ*l of 10 mg/mL solution of each of the compounds was added to the wells of the 1st and 2nd column. From the 2nd column, serial dilutions were performed yielding final concentrations of 500; 250; 125; 62.5; 31.2; and 15.6 *μ*g/mL for each of the compounds. The volume was completed with 100 *μ*L bacterial culture completing a final volume of 200 *μ*L. The plates and tubes were incubated at 37°C for 24 hours for reading, performed by addition of 20 *μ*L of a 0.01% (w/v) Resazurin sodium (Sigma) solution, a colorimetric indicator of metabolic activity [26].

### 2.4. Molecular Docking

To predict the binding interactions the isolated compounds in the 14*α*-demethylase binding site [27] on the basis of Minimum Inhibitory Concentration (MIC) values and molecular docking (MD) were conducted using the MOEDock program. The crystal structure of 14*α*-demethylase (PDB ID 3LD6) was retrieved from the protein data bank. Prior to MD, 3D protonation and energy minimization (EM) of the crystal structures were analyzed using the default parameters of the MOE energy minimization algorithm (gradient: 0.05, Force Field: Amber99) [28]. All of the synthesized compounds were categorized based on the MIC value; categories 01, 02, 03, and 04 were standardized as MIC values of less than 1, 2, and 3 and nonactive. Using the Molecular Builder Module program implemented in MOE, 3D structures of all the compounds were constructed and saved in the (mdb) file format for MD. Subsequently, EM for all of the compounds was carried out up to the 0.05 Gradient using MMFF94s force field implemented in MOE. All of the compounds were docked into the active site of the protein using the Triangular Matching docking method (default) and five different conformations for each compound (as allowed to be generated). The predicted ligand-protein complexes were ranked by the scores of the GBVI/WSA free binding energy (FBE) calculation. GBVI/WSA is a scoring function which estimates the FBE of the ligand in a given pose. For scoring functions, lower scores indicate the more favorable mode of interaction. The unit for scoring functions is kcal/mol. The top-ranked conformation of each compound was selected on the basis of these docking scores (S) for further analysis. At the end of docking, the predicted ligand-protein complexes were analyzed for molecular interactions in PyMol [29].

## 3. Results and Discussion

**Scheme 1:**
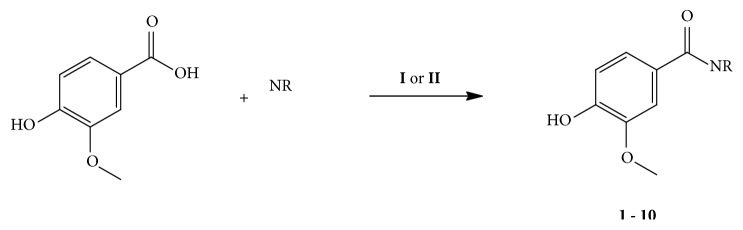
Preparation of amides derived from vanillic acid (**1-10**).** I**: DCC, DMAP, CH_2_Cl_2_, t.a.;** II:** DMF, Et_3_N, PyBOP, CH_2_Cl_2_, 0°C to t.a.

Vanillic acid was used as the starting material for the preparation of a collection of ten amides that were obtained through coupling reaction with PyBOP (**1-6**) and through coupling reaction with DCC (**7-10**) using the amines isobutylamine, pyrrolidine, cyclohexylamine, aniline, benzylamine, 4-fluorobenzilamine, 4-methylbenzylamine, 4-methoxybenzylamine, 3.4-dimethoxybenzylamine, and piperonylamine (Scheme 1).

**Figure 2:**
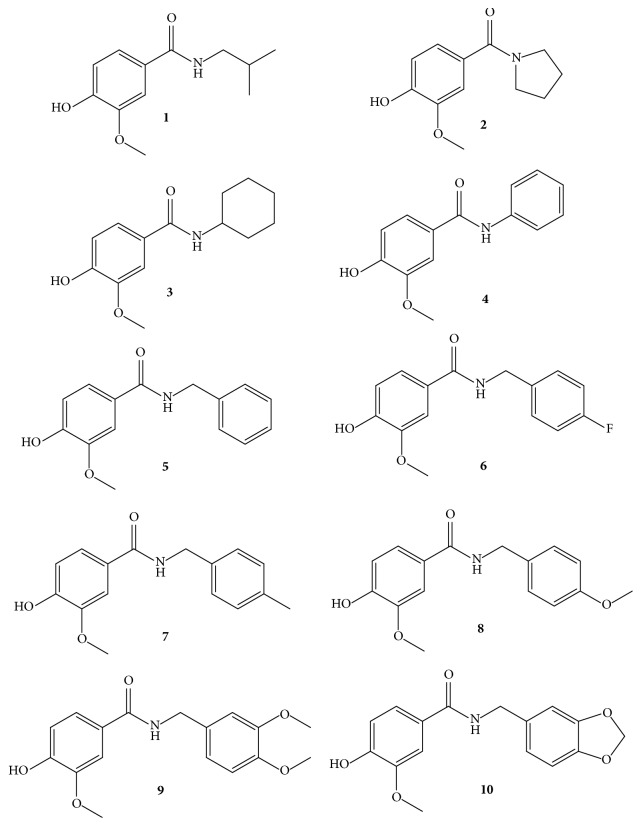
Vanillic acid amide analogs** 1-10**.

In ^1^H NMR analysis, the guiding signals in common for all of the amides derived from vanillic acid (Figure 2) were aromatic hydrogens as two doublets and a double doublet around 7.44 (*d*,* J =* 2.0 Hz, 1H), 6.79 (*d*,* J = *8.2 Hz, 1H), and 7.38 (*dd*,* J =* 8.3, 2.1 Hz, 1H) allocated to hydrogens H-2, H-5, and H-6, respectively. The similar coupling constants demonstrate coupling that occurs between the hydrogens of the aromatic ring. Thus, H-6 engages the neighboring hydrogen (H-5) but also features four coupling links with H-2, which is common in aromatic systems. Compound** 1** presented signals of an aliphatic side-chain, at 3.27 (*t*,* J =* 6.2 Hz, 2H) of methylene hydrogen (H-1′), 1.88 (*m*, Hz, 1H) of methine hydrogen (H-2), and 0.97 (*d*,* J =* 6.7 Hz, 6H) for the two methyls (H-3′ and 4′) of the isobutyl substituent. Compound** 2** presented signals of a cyclical side chain (a 5 member ring) at 3.61 (*t*,* J =* 5.7 Hz, 2H, H-1′), 1.96-1.80 (*m*, 4H, H-3′ and H-4′), and 3.48 (*t*,* J =* 5.4 Hz, 2H, H-2′) of methylene hydrogens. Compound** 3** presented signals of a cyclohexane ring at 1.73-1.41 (*m*, 6H, H-2′, H-4′, H-6′) and 1.24-1.11 (*m*, 4H, H-3′, H-5′) of methylene hydrogens. Compound** 4** presented signals related to hydrogens of an aromatic system at 7.63 (*dd*,* J =* 8.6, 1.2 Hz, 2H, H-2′, H-6′), 7.36 (*dt*,* J =* 3.0, 1.9 Hz, 2H, H-3′, H-5′), and 7.16-7.10 (*m*, 1H, H-4′). Compound** 5** presented signals related to an aromatic system and methylene hydrogens (NH-C**H**_**2**_) at 7.34 (*d*,* J =* 4.4 Hz, 4H, H-2′, H-3′, H-5′, H-6′), 7.30-7.27 (m, 1H, H-4′), and 4.61 (*d*,* J =* 5.7 Hz, 2H, H-7′), respectively. Compounds** 6, 7,** and** 8** presented signals related to an aromatic system and methylene hydrogens similar to compound** 5**, if differing by the presence of a substitution in the* para* position of the aromatic ring. Compound** 6** only presented signals of an aromatic ring and of methylene hydrogens due to the fluoride substituent. Compound** 7** presented signals of methyl hydrogen (CH_**3**_) at 2.32 (*s*, 3H). Compound** 8** presented signals of methoxyl hydrogen (OCH_3_) at 3.81 (*s*, 3H). Compound** 9** presented signals of an aromatic system and methylene hydrogens at 6.88 (*q*,* J =* 6.2, 2.1 Hz, 3H, H-2′, H-3′, H-6′) and 4.54 (d, J = 5.6 Hz, 2H, H-7′), respectively, as well as two methoxyl signals at 3.84 (*d*,* J =* 3.1 Hz, 6H) in the* meta* and* para* positions of the aromatic ring. Compound** 10** presented signals of methylene hydrogen (H-8′) belonging to the dioxy-methylene substituent at 5.90 (*s*, 2H), along with signals of aromatic hydrogens and methylene hydrogen. The guiding signals in common to all of the amides in the ^13^C NMR spectra were around 166.05, characteristic of amide C=O, two signals at 149.53 and 147.25 assigned, respectively, to carbon C-4 and C-3, and signals at around 125.23, 120.81, 115.01, and 111.33 relating, respectively, to carbon C-1, C-6, C-5, and C-2. Compound** 1** presented signals of methylene, methine, and methyl carbon, respectively, at 47.32 (C-1′), 28.44 (C-2), and 20.34 (C-3′, C-4′). Compound** 2** presented signals of methylene carbons (5 member ring) at 49.89 (C-1′), 46.30 (C-2′), 26.32 (C-3′), and 24.26 (C-4′). Compound** 3** presented signals of cyclohexane ring methylene carbons at 48.10 (C-1′), 32.40 (C-2′, C-6′), 25.13 (C-4′), and 24.86 (C-3′ C5′). Compound** 4** presented signals of aromatic ring carbons at 138.20 (C-1′), 129.88 (C-6′), 129.17 (C-3′, C-5′), and 124.53 (C-2′, C-4′). Compound** 5** presented signals of methylene carbon and aromatic system carbons at 44.27 (C-7′), 138.48 (C-1′), 128.87 (C-3′), 127.99 (C-5′), 127.90 (C-2′), 127.80 (C-6′), and 127.67 (C-4′). Compounds** 6**,** 7**, and** 8** presented signals for aromatic and methylene carbons similar to compound** 5**, differing by the presence of a* para* substitution in the aromatic ring. Compound** 6** presented a signal at 161.25 (*d*, ^*1*^*J*_*C*-*F*_ = 237.5 Hz, C-4′-F), assigned to** C**-F coupling. Compound** 7** presented a** C**H_3_ carbon signal at 21.17. Compound** 8** presented an OCH_3_ carbon signal at 55.28. Compound** 9** presented two OCH_3_ carbon signals at the* meta* and* para* positions of the aromatic ring at 56.08 and 55.08. Compound** 10** presented a methylene carbon signal of the dioxy-methylene substituent at 101.18 (C-8′).

In this study ten amides were tested against strains of* Candida* (Table 1). In accordance with the MFC/MIC relation [30] and as contained in Table 1, all of the tested compounds presented fungicidal activity against the tested strains. The amides,** 1 3, 5,** and** 8,** were active against the strain of* Candida albicans* at the highest concentration tested, while the amides** 4** and** 6** were bioactives, presenting respective MICs of 2.17 and 1.81 *μ*mol/mL. These data are in agreement with a previous study in which it tested several bioactive halogenated amides against bacterial and fungal strains, among them the vanillic amide N-(4-fluorobenzyl)-4-hydroxy-3-methoxybenzamide, which presented a MIC of 1.81 *μ*mol/mL against a* Candida albicans* strain [13]. The other amides showed no activity against this strain. It can be suggested that for amide** 2** in relation to amide** 3** the pyrrolidine ring connected directly to the nitrogen in amide** 2** generates inactivity against the* C. albicans* strain, which is different from amide** 3** that presents a (nonheterocyclic) cyclohexane ring. The presence of an alkyl group (isobutyl group) demonstrated in amide** 1** contributed to bioactivity against the* C. albicans* strain. Comparing amide** 3** with amide** 5 **suggests that the presence of the aromatic ring in amide** 5** caused no significant change in the MIC. When comparing amides** 7, 8, 9,** and** 10**, it is likely that the methoxyl attached to the aromatic ring in the* para* position contributed to amide** 8** antifungal activity. The presence of* para* methyl substituents (**7**), dioxymethylene (**10**), or di-methoxyl (**9**) may influence the compounds inactivity. The presence of the methoxyl in the* para* position of compound** 8** results in inactivity against the* C. albicans* strain. Analyzing amide** 3** (MIC = 4.00 *μ*mol/mL), together with amide** 4** (MIC of 2.17 *μ*mol/mL), it can be inferred that aromaticity resulted in an amide** 4** MIC decrease, as compared to the cyclohexane present in amide** 3**. While in amide** 6**, it was observed that the presence of an electron removing group such as fluoride in the aromatic ring caused an MIC decrease to 1.81 *μ*mol/mL and the consequent improvement of activity in relation to the ring* para*-methyl (donor group) in amide** 7** against the* C. albicans* strain. De Vita et al. (2014) tested compounds with a methoxyl substituent at the* para* position and a fluoride at the* meta*-position presenting antifungal activity against of strains of* C. albicans* [31]. The data indicated that fluoride contributes to compound bioactivity. In the test using the* Candida glabrata *strain, the bioactive amides were** 4**,** 6,** and** 7**. The amides** 6** and** 7** presented activity in the highest concentration tested (MIC = 3.62 and 3.68 *μ*mol/mL) yet not presenting differences in potency with the changes of the* para* position substituent on the benzene ring. Compound** 4** presented an MIC = 2.17 *μ*mol/mL, showing that the presence of the benzene ring linked directly to the nitrogen contributes to improvement of activity, being the same as with the* C. albicans* strain. In the test using* Candida krusei*, only amides** 4** and** 6** were bioactive presenting MICs for the concentrated tested. We note that the presence of substituents on both amides contributed to bioactivity against the four strains tested. Another strain tested was* Candida guilliermondii*, which presented sensitivity to a greater number of compounds in relation to the other strains. The amides** 1-5** presented MICs of from 2.25 *μ*mol/mL to 1.94 *μ*mol/mL. These results suggest that changing the substituent group on these amides did not cause significant changes in bioactivity against the* C. guilliermondii* strain. The amides** 9** and** 10** presented respective MICs of 1.66 *μ*mol/mL and 1.57 *μ*mol/mL, indicating that varying substituents did not potentiate compound bioactivity. Amide** 7** was bioactive with an MIC value of 0.46 *μ*mol/mL, thus presenting the greatest activity in relation to the other amides. One can again suggest that the presence of an electron donor group attached to the aromatic ring in the* para* position (such as a methyl) can generate an increase in antifungal potency against the* C. guilliermondii* strain. A study developed by Reddy, Ravinder, and Kanjinal (2012) has demonstrated that vanillic derivatives have antifungal activity against species of the genus* Candida* [32].

The most outstanding amide was compound** 7** with an MIC = 0.46 *μ*mol/mL against the* Candida* strains. This compound was then subjected to mechanism of action testing against the* C. guilliermondii* strain via two pharmacological strategies, respectively, using ergosterol and sorbitol to determining the likely activity in the plasma membrane or cell wall (Table 2). Sterols participate in the constitution of all types of fungal cells. Ergosterol is the principal sterol and acts by modulating membrane fluidity, growth, and cell proliferation [33, 34]. Testing to detect the biological target of the amide** 7** was performed by adding more ergosterol to the medium containing the fungus and the compound. There was an increase in the MIC of the compound to 3.68 *μ*mol/mL, indicating that the fungus used the added ergosterol to reproduce, which demonstrates that the compound acts possibly by inhibiting synthesis or through binding directly to ergosterol. Azoles and polyenes are antifungal drug classes that act on ergosterol to treat fungal infections [4]. In the test using sorbitol, an osmotic protector, which acts by preventing changes in the fungal cell wall, the MIC of amide** 7** increased, permitting growth at all concentrations, indicating that the substance acts by interfering with cell wall synthesis as well. Without the osmotic protector, the compound would cause lysis of the cells in the cell wall, yet in the presence of sorbitol, the fungus is protected and continues to reproduce. The substance thus acts through interference with the permeability of the cell membrane, with action on ergosterol, and in modulating cell wall function [35].

All tested compounds presented antibacterial activity against the Gram-positive lineage strain* Staphylococcus aureus* ATCC 25925, as evidenced by the minimum inhibitory concentration (MIC), the highest concentration tested for all the amides prevailed (Table 3). Amides** 8, 9,** and** 10** presented better antibacterial activities (with MIC values of between 3.14-3.48 *μ*mol/mL), as compared to the other amides tested, which presented no bioactivity with changing of substituents. Thus, it can be suggested that the presence of one and/or two methoxyl group substituents in the* meta* and* para* positions or a dioxymethylene group in the compound enhances its antibacterial action.

**Figure 3:**
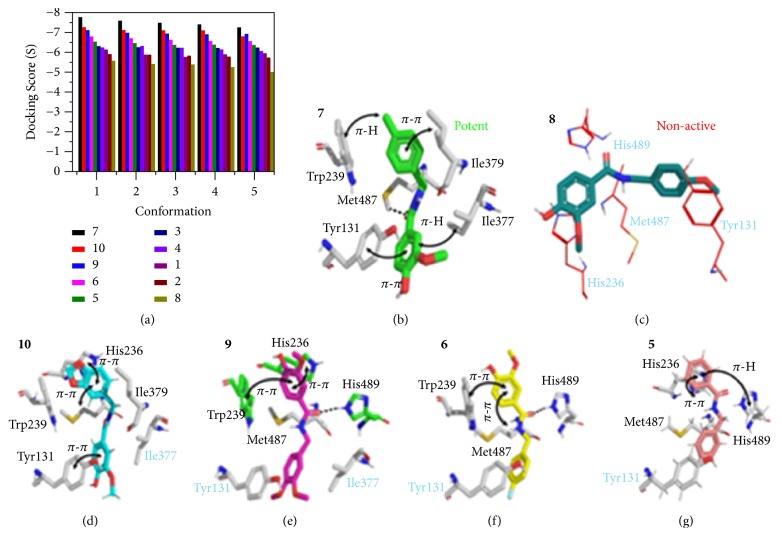
The molecular interactions and surface visualization of the synthesized compounds in the 14*α*-demethylase enzyme active site (PDB code 3LD6). (a) Best predicted conformers based on docking score (S), (b) most active compound** 7**, (c) inactive compound** 8**, and (d–g) category-2 compounds. The ligand is shown in a different color for each pose.

Top conformers, based on docking scores (Figure 3(a)), were further selected for ligand-protein interaction analysis in the active cavity of the target protein. From the MD study, it was observed that, for all compounds, the pi terminal electron systems of the aromatic rings presented molecular interactions with the active site residues. All of the synthesized compounds were categorized based on MIC values. Category-1 included only one, the most active or potent compound, amide** 7** (Figure 3(b)); category-4 included the nonactive amide** 8** (Figure 3(c)). Comparing the modes of interaction of both** 7** and** 8**, five interactions with active site residues were adopt by compound** 7**. Tyr131 and Ile379, respectively, form pi-pi and pi-H interactions with the terminal aromatic rings of the compound. Similarly, Ile377 and Trp 239 established pi-hydrogen interactions. Met487 forms a hydrogen bond (2.1Å) with the compound's formamide oxygen. For compound** 8**, no interactions were observed with the active site residues reflecting its nonactive nature. This might be caused by polar groups of the compound (attached to the terminal phenyl rings) which restrict the compound from molecular interaction. Category-2 included amides** 10**,** 9**,** 6**,** 5**, and** 3**. All of the compounds in this category adopted various pi-pi and hydrogen interactions. Most of the interactions observed were pi-pi and hydrogen bonds with the compound's side benzo[*d*][1,3] dioxol, methoxyphenol, and formamide moiety oxygen. Amide** 10** pi-pi interactions were observed with the active site residues His236, Trp 239, and Tyr131 as seen in Figure 3(d). For compounds** 9**,** 8**, and** 5** as presented in Figures 3(e), 3(f), and 3(g), similar interaction modes were observed, with the exception of His489 that was found to be involved in hydrogen bonding (with compounds** 9** and** 6**) and a pi-H bond with compound** 5**. All the blue colored label residues indicate active site residues with no interactions. Category-3 includes amides** 4**,** 1**, and** 2**, which were generally observed making only hydrophobic interactions (data not shown), which stabilized the compounds in the active site cavity and make these compounds active against the target protein. On the whole for these compounds, the docking study revealed good correlations with the biological study.

## 4. Conclusion

All of the amides tested presented antifungal activity in against most of the strains tested. Amide** 7** presented the best activity (lowest MIC) against* C. guilliermondii* strain 207, suggesting that the presence of a methyl group in the* para* position of the aromatic ring helps to potentiate antifungal activity. The remaining compounds presented low to moderate to bioactivity against the tested strains. The activity of amide** 7** in fungal species suggests modification of cell wall functions and the plasma membrane. In the antibacterial tests, only* Staphylococcus aureus* strain ATCC 25925 presented sensitivity to all of the compounds. From molecular docking carried out with the ten derived compounds, a better interaction for compound** 7** with 14*α*-demethylase was observed, suggesting it as a biological target. Thus, by presenting promising new prototypes, the results explained in this work serve as a basis for further research involving more powerful antimicrobials using vanillic acid derived amides.

## Figures and Tables

**Table 1:** Minimum inhibitory concentration (MIC), maximum fungicidal concentration (MFC) values, and MFC/MIC in *μ*mol/mL in amides (**1-10**) against strains of the genus *Candida*.

Amides	*Candida albicans*			*Candida glabrata*			*Candida krusei*			*Candida guilliermondii*		
	(ATCC 90028)			(ATCC 90030)			(ATCC 34125)			(207)		
	MIC	MFC	MFC/MIC	MIC	MFC	MFC/MIC	MIC	MFC	MFC/MIC	MIC	MFC	MFC/MIC
**1**	4.48	4.48	1	+	+	+	+	+	+	2.24	2.24	1
**2**	+	+	+	+	+	+	+	+	+	2.25	2.25	1
**3**	4.00	4.00	1	+	+	+	+	+	+	2.00	2.00	1
**4**	2.17	2.17	1	2.17	2.17	1	4.34	4.34	1	2.17	2.17	1
**5**	3.88	3.88	1	+	+	+	+	+	+	1.94	1.94	1
**6**	1.81	1.81	1	3.62	3.62	1	3.62	3.62	1	1.81	1.81	1
**7**	+	+	+	3.68	3.68	1	+	+	+	0.46	0.46	1
**8**	3.48	3.48	1	+	+	+	+	+	+	+	+	+
**9**	+	+	+	+	+	+	+	+	+	1.66	1.66	1
**10**	+	+	+	+	+	+	+	+	+	1.57	1.57	1
**Control medium**	-	-	-	-	-	-	-	-	-	-	-	-
**Nistatin**	0.00043	0.00043	0.00043	0.00043	0.00043	0.00043	0.00043	0.00043	0.00043	0.00043	0.00043	0.00043
**Control microorganism**	+	+	+	+	+	+	+	+	+	+	+	+

+: microorganism growth. -: no microorganism growth.

**Table 2:** Effects of amide **7** MIC against *C. guilliermondii*, in the presence or absence of a protective osmotic (sorbitol 0.8 M) as well as in the presence or absence of ergosterol.

Concentration (*μ*mol/mL)	*Candida guilliermondii*			
	(207)			
	No sorbitol	With sorbitol	No ergosterol	With ergosterol
3.68	-	+	-	-
1.84	-	+	-	+
0.92	-	+	-	+
0.46	-	+	-	+
0.23	+	+	+	+
0.11	+	+	+	+
Caspofungin	0.3 *μ*mol/mL	4.5 *μ*mol/mL	-	
Nistatin	-		0.00043 *μ*mol/mL	0.26 *μ*mol/mL

+:microorganism growth. -: no microorganism growth.

**Table 3:** Bioactivity of the amides prepared against quantified strains of bacteria through the minimum inhibitory concentration (MIC) in *μ*mol/mL.

Amides	*Pseudomonas aeruginosa *	*Pseudomonas aeruginosa *	*Staphylococcus aureus *	*Staphylococcus aureus*
	(ATCC 8027)	(102)	(ATCC 25925)	(47)
**1**	+	+	4.48	+
**2**	+	+	4.50	+
**3**	+	+	4.01	+
**4**	+	+	4.34	+
**5**	+	+	3.88	+
**6**	+	+	3.62	+
**7**	+	+	3.68	+
**8**	+	+	3.48	+
**9**	+	+	3.32	+
**10**	+	+	3.14	+
**Control medium**	-	-	-	-
**Chloramphenicol**	0.3095	0.3095	0.3095	0.3095
**Control microorganism**	+	+	+	+

+:microorganism growth. -: no microorganism growth.

## Data Availability

The article data used to support the findings of this study have been deposited in the Federal University of Paraíba repository https://repositorio.ufpb.br/jspui/handle/123456789/13651.
